# Extracellular microRNAs are dynamic non-vesicular biomarkers of muscle turnover

**DOI:** 10.1093/nar/gkt724

**Published:** 2013-08-14

**Authors:** Thomas C. Roberts, Caroline Godfrey, Graham McClorey, Pieter Vader, Deborah Briggs, Chris Gardiner, Yoshitsugu Aoki, Ian Sargent, Jennifer E. Morgan, Matthew J.A. Wood

**Affiliations:** ^1^Department of Physiology, Anatomy and Genetics, University of Oxford, South Parks Road, Oxford, OX1 3QX, UK, ^2^Department of Molecular and Experimental Medicine, The Scripps Research Institute, 10550 N. Torrey Pines Road, La Jolla, CA 92037, USA, ^3^Dubowitz Neuromuscular Centre, UCL Institute of Child Health, 30 Guildford Street, London, WC1N 1EH, UK and ^4^Nuffield Department of Obstetrics and Gynaecology, University of Oxford, Level 3, Women's Centre, John Radcliffe Hospital, Oxford, OX3 9DU, UK

## Abstract

Extracellular microRNAs (miRNAs) are promising biomarkers of the inherited muscle wasting condition Duchenne muscular dystrophy, as they allow non-invasive monitoring of either disease progression or response to therapy. In this study, serum miRNA profiling reveals a distinct extracellular miRNA signature in dystrophin-deficient *mdx* mice, which shows profound dose-responsive restoration following dystrophin rescue. Extracellular dystrophy-associated miRNAs (dystromiRs) show dynamic patterns of expression that mirror the progression of muscle pathology in *mdx* mice. Expression of the myogenic miRNA, miR-206 and the myogenic transcription factor myogenin in the tibialis anterior muscle were found to positively correlate with serum dystromiR levels, suggesting that extracellular miRNAs are indicators of the regenerative status of the musculature. Similarly, extracellular dystromiRs were elevated following experimentally-induced skeletal muscle injury and regeneration in non-dystrophic mice. Only a minority of serum dystromiRs were found in extracellular vesicles, whereas the majority were protected from serum nucleases by association with protein/lipoprotein complexes. In conclusion, extracellular miRNAs are dynamic indices of pathophysiological processes in skeletal muscle.

## INTRODUCTION

The progressive muscle wasting condition Duchenne muscular dystrophy (DMD) is caused by loss-of-function mutations in the *DMD* gene, which encodes the structural and signalling protein dystrophin (1). The dystrophin gene consists of 79 exons, many of which encode redundant structural domains. DMD is therefore amenable to molecular correction by antisense oligonucleotide-mediated exon skipping therapy in which a specific exon is preferentially excluded by the splicing machinery to produce an internally deleted yet partially functional dystrophin protein, thereby achieving molecular correction of the disease (2). Exon skipping therapy for DMD shows promise in pre-clinical animal models (3) and in clinical trials in patients (4–6). However, current molecular assessment of dystrophin in patients requires invasive muscle biopsy. Consequently, there is a need to develop non-invasive biomarkers for monitoring disease progression and response to novel therapeutics.

MicroRNAs (miRNAs) are small RNA species (∼22 nt long) that are endogenous RNA interference effectors and act primarily as post-transcriptional regulators of gene expression (7). miRNAs have been implicated in the control of a wide variety of cellular processes and disease conditions, including DMD (8). Recently, it has become apparent that miRNAs are readily detectable in bodily fluids including serum, plasma, urine, cerebral spinal fluid, saliva and seminal fluid (9). Given their remarkable nuclease stability in the extracellular environment, miRNAs are of interest as potential disease biomarkers (10,11). Additionally, given that miRNAs are regulators of physiological processes, their abundance in bodily fluids may reflect their expression in their cells or tissues of origin.

We (12), and others (13,14), have shown that the serum of dystrophic animal models (*mdx* mouse and CXMD_J_ dog) and DMD patients is enriched for the dystrophy-associated miRNAs (dystromiRs): miR-1, miR-133 and miR-206. Furthermore, extracellular dystromiR levels are restored by exon-skipping therapy using small nuclear RNA (snRNA) expression constructs (13,15) and Peptide-Phosphorodiamidate Morpholino Oligonucleotide (PPMO) conjugates (12) in dystrophin-deficient mouse models (*mdx* and *dko*). We have previously shown that the miRNA profile of serum shows little similarity with that of muscle in the *mdx* mouse and proposed that this might be explained by selective release, rather than passive leakage, of miRNAs from dystrophic muscle (12).

An understanding of the ontology of serum miRNA biomarkers will be crucial for their accurate clinical interpretation. Here, we provide an in-depth analysis of extracellular dystromiRs in the *mdx* mouse to shed light on their biological and clinical significance. We have shown differential restoration of serum dystromiRs in response to varying levels of dystrophin rescue, identified novel biomarkers by serum miRNA profiling and demonstrated that dystromiRs are protected from serum nucleases through association with proteins/lipoproteins. By analysing serum dystromiR abundance over time, we show that the levels of circulating miRNAs follow the development of the underlying muscle pathology in the *mdx* mouse. We conclude that serum dystromiRs are dynamic non-vesicular biomarkers of muscle turnover.

## MATERIALS AND METHODS

### Animal procedures

Animal experiments were carried out in accordance with the Animals (Scientific Procedures) Act 1986. PPMO conjugates were prepared as described previously (3). In all experiments, 12.5 mg/kg of PPMO was administered via the tail vein of 12-week-old male *mdx* mice under isoflurane anaesthesia and animals sacrificed at various time points. For the muscle injury study, 8-week-old male C57Bl/10 mice were anaesthetized with isoflurane, and 25 µl of 10^−^^5 ^M cardiotoxin (CTX) (Latoxan, Valence, France) was injected per-cutaneously into the tibialis anterior (TA) muscles of the right and left hindlimbs. TA muscles were removed 14 days after CTX injection, and 7 μm sections stained with Hematoxylin and Eosin for histological analysis. The number of centrally nucleated fibres (CNFs) was counted on three representative serial sections.

### RT-qPCR

Serum was extracted post-mortem using Microvette CB300 capillary serum collection tubes (Sarstedt Ltd, Leicester, UK), and RNA was extracted from 50 μl of serum using TRIzol LS (Invitrogen, Paisley, UK) as according to manufacturer’s instructions. A synthetic miRNA, cel-miR-39, was added as a normalization control at the organic extraction phase. miRNAs were reverse transcribed and quantified by small RNA TaqMan Reverse Transcriptase-quantitative Polymerase Chain Reaction (RT-qPCR) normalized to cel-miR-39 levels. All primer/probe assays were purchased from Applied Biosystems (Warrington, UK). Where appropriate, serum samples were treated with either 1 mg/ml Proteinase K (Roche, San Francisco, CA) at 55°C or 1% Triton X-100 (Sigma, Dorset, UK), and aliquots were sampled at specific time points.

Mouse TA muscle, diaphragm and heart were macrodissected, snap-frozen in liquid nitrogen-cooled isopentane, homogenized using a Precellys 24 (Bertin Technologies, Paris, France) and RNA extracted using TRIzol reagent (Invitrogen) as according to manufacturer’s instructions. RNA was reverse transcribed and miRNA/mRNA expression determined by RT-qPCR. All qPCR reactions were performed on a StepOne Plus real-time thermocycler (Applied Biosystems). All primer and probe sequences are shown in Supplementary Table S2. Gene-of-interest expression levels were normalized to the geometric mean of four reference genes (Actb, Tbp, Rplp0 and Rpl10). Relative quantification analysis was performed using the Pfaffl method (16). Raw Ct data are provided in Supplementary Data File S1.

### Serum miRNA profiling

Serum miRNA profiling was performed on the miRCURY LNA SYBR Green RT-qPCR array platform by Exiqon Services (Copenhagen, Denmark). Total RNA was extracted from serum using the Qiagen (Crawley, UK) miRNeasy® Mini Kit. Fifteen microlitres of RNA was reverse transcribed in 75 μl of reactions using the miRCURY LNA™ Universal RT microRNA PCR, Polyadenylation and cDNA synthesis kit (Exiqon). cDNA was diluted 1 in 50 and assayed in 10 μl of PCR reactions according to the protocol for miRCURY LNA™ Universal RT microRNA PCR; each miRNA was assayed once by qPCR on the miRNA Ready-to-Use PCR, Rodent panel I and panel II. Additionally, negative controls (no template) were analysed in parallel. RT-qPCR was performed in a LightCycler® 480 Real-Time PCR System (Roche) in 384-well format. The amplification curves were analysed using the Roche LC software, both for determination of Ct (by the second derivative method) and for melting curve analysis. The best normalizer was found to be the average of assays detected in all samples using the NormFinder algorithm (17). All data were normalized to the average of assays detected in all samples [average (*n* = 12) – assay Ct] as described previously (18).

Given that SYBR green-based methods of template detection are intrinsically prone to artifacts, especially at low template copy numbers, stringent quality control measures were applied to the array data. Data points were excluded if multiple melt peaks or atypical melting temperatures were observed. PCR efficiencies were estimated using the LinReg algorithm as described previously (19,20) with all reactions having efficiencies in the range of 80–110% included in the analysis. Assays with signal detected on the no template control plate within 5 Ct values of the values detected on the sample plates were also excluded. External spike-in oligonucleotides amplified with similar Ct values across all samples indicating equivalent RNA extraction and RT efficiencies (Supplementary Figure S5). Furthermore, the Ct values were similar in the no template control sample, suggesting that PCR inhibitors were not co-purified with serum RNA. Haemolysis was assessed by visual inspection and by measuring the ratio of miR-451 to miR-23a abundance. (miR-451 is an erythrocyte-enriched miRNA, whereas miR-23a is a highly expressed and stable in serum and unaffected by haemolysis) (21). No sample appeared visibly haemolyzed (Supplementary Figure S6A) or had a miR-451/miR-23a ratio that exceeded eight, which was the pre-determined quality control cut-off (Supplementary Figure S6B), suggesting that miRNAs derived from lysed erythrocytes were unlikely to affect the results. Raw data are provided in Supplementary Data File S1.

### Isolation of extracellular vesicles

Extracellular vesicles (EVs) were isolated by one of three methods: (i) centrifugation at 30 000 *g* for 1 h, (ii) ultra-centrifugation at 100 000 *g* for 1 h or (iii) ultra-filtration using 1 MDa Vivaspin filters (Sartorius, Stedim Biotech GmbH, Goettingen, Germany). Particle count and size distribution of EVs were assessed by Nanoparticle Tracking Analysis (NTA) using a Nanosight NS500 (Nanosight Ltd, Amesbury, UK). Three videos of 60 s were recorded for each sample. Brightness and gain settings were standardized across samples, and concentration measurements were standardized as previously described (22). Videos were analysed using NTA software (version 2.3) with the minimal expected particle size, minimum track length, blur and detection threshold all set to automatic.

### Dystrophin protein quantification

Dystrophin western blotting was performed using the XCell SureLock System (Invitrogen) and the following antibodies: NCL-DYS1 mouse monoclonal antibody, (Leica Biosystems, Newcastle, UK), the loading control vinculin hVIN-1 mouse monoclonal antibody (Sigma-Aldrich) and a secondary antibody IRDye 800CW Goat anti-Mouse (LiCOR Biosciences, Lincoln, NE). Band intensities were visualized and quantified using the Odyssey imaging system (LiCOR Biosciences). Quantitative immunofluorescence was performed using the following antibodies: dystrophin (ab15277, Abcam, Cambridge, UK) and Laminin α-2 Chain (L0663, Sigma) and secondary antibodies Alexa Fluor® 594 (goat anti-rabbit IgG) and Alexa Fluor® 488 (goat anti-rat IgG) (both Invitrogen). Immunohistological intensity measurements were performed as previously described using ImagePro software (Media Cybernetics, Rockville, MD) (3,23,).

### miRNA immunoprecipitation

Hundred microlitre serum samples were diluted in phosphate buffered saline and immunoprecipitated with 5 μg of one of the following antibodies: anti-Ago2 (ab32381, AbCam), anti-ApoA-1 (sc-30089, Santa Cruz Biotechnology, Inc., Dallas, TX) or anti-IgG (PP64B, Merck-Millipore, Billerica, MA). Immune complexes were pulled down using 50 μl of Protein G magnetic Dynabeads (Invitrogen). The beads were collected using a magnet and washed three times with miRIP wash buffer [50 mM Tris–HCl (pH 7.4), 1% NP-40, 150 mM NaCl, 2 mM EDTA] and resuspended in 100 μl of RNase free phosphate buffered saline. RNA was extracted using TRIzol LS as described earlier in the text.

### Statistical analysis

Two sample comparisons were tested for statistical significance using a two-tailed Student’s *t*-test. For comparisons of more than two groups, one-way analysis of variance (ANOVA) and Bonferroni correction *post hoc* test were performed using GraphPad Prism 5 (GraphPad Software Inc, La Jolla, CA). Spearman correlation analyses were also performed using GraphPad Prism 5. Differences were considered significant at *P*-values below 0.05. Hierarchical clustering analysis, ANOVA of array data, principal component analysis and heatmaps were produced using MeV (Multiple Experiment Viewer) (Institute for Genomic Research, Rockville, MD) (24).

## RESULTS

### Dose-responsive restoration of serum dystromiRs following therapeutic intervention

**Figure 1.**
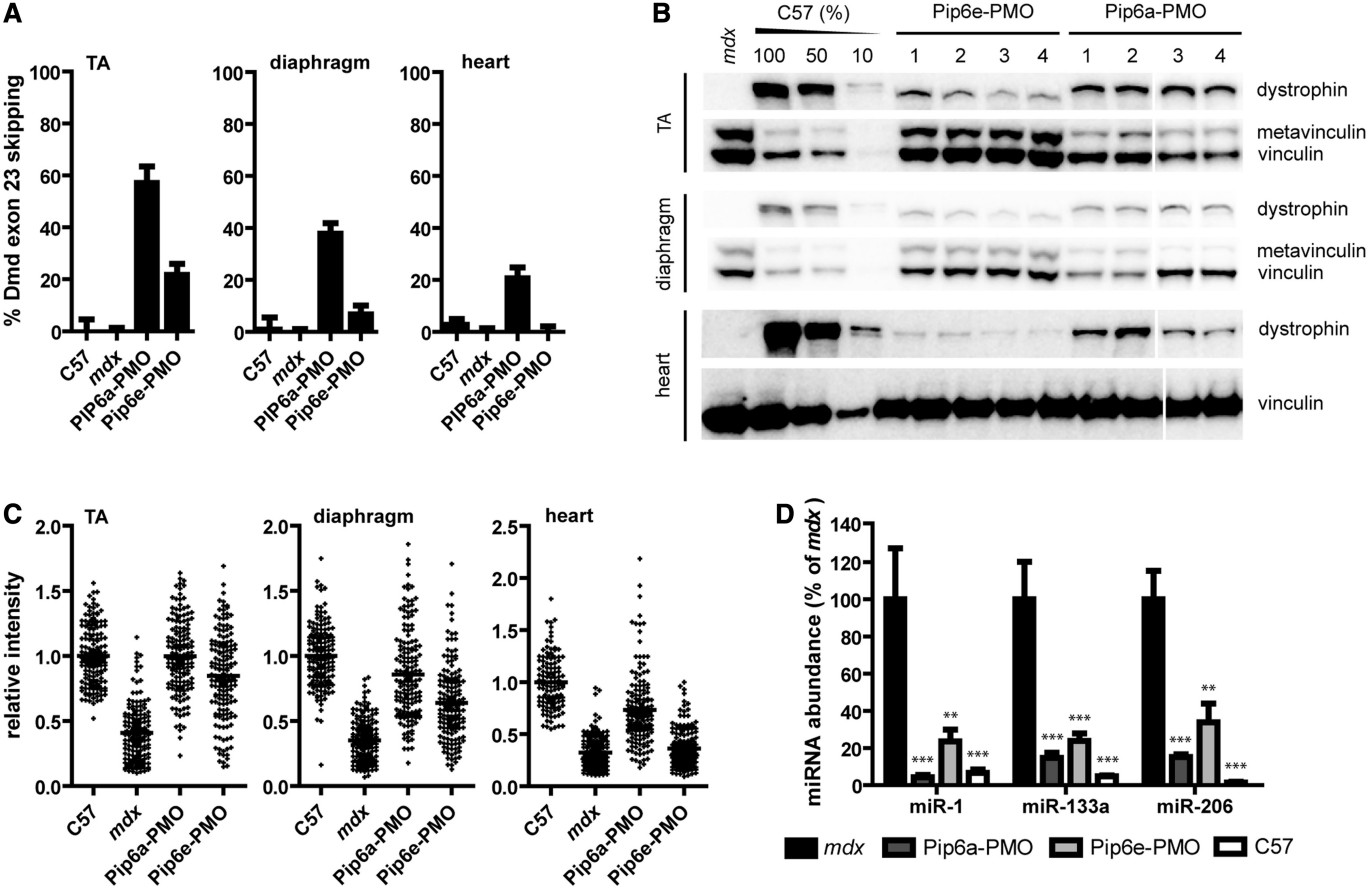
Restoration of circulating dystromiR by PPMO conjugates. The 12-week-old male *mdx* mice were treated with a single intravenous 12.5 mg/kg dose of either Pip6a-PMO or Pip6e-PMO. Mice were sacrificed 2 weeks post-injection along with age-matched C57Bl/10 and *mdx* controls. The TA, diaphragm and heart muscles were dissected, and serum was harvested. Treatment with PPMO conjugates induces (**A**) exon skipping of the Dmd pre-mRNA as determined by RT-qPCR and dystrophin protein restoration as determined by (**B**) western blotting and (**C**) quantitative immunofluorescence. (Western blots are composites of two gels as indicated by break.) In the case of all assays, Pip6a-PMO is more effective at inducing exon skipping and dystrophin protein restoration than Pip6e-PMO and is also active in the heart. (**D**) Serum dystromiR (i.e. miR-1, miR-133a and miR-206) levels were determined by small RNA TaqMan RT-qPCR assays normalized to an external spike-in control. Pip6a-PMO treatment induces greater dystromiR restoration than Pip6e-PMO. Values are mean + SEM, **P < 0.01, ***P < 0.001, *n* = 4.

We have previously shown that systemic treatment of *mdx* mice with PPMO conjugates can partially restore circulating dystromiR abundance towards wild-type levels (12). Having further optimized this approach, we sought to compare the effect on miRNA restoration when using a more effective PPMO. To this end, we assessed dystrophin restoration by two different PPMO conjugates (Pip6a-PMO and Pip6e-PMO). Both conjugates induced high levels of exon skipping and protein restoration in peripheral muscle after a single intravenous 12.5 mg/kg administration. However, Pip6a-PMO was found to be more effective at restoring dystrophin as determined by RT-qPCR detection of skipped Dmd transcripts (Figure 1A), western blot (Figure 1B) and quantitative immunofluorescence (Figure 1C).

Circulating miRNA levels were determined by small RNA TaqMan RT-qPCR in the Pip6a-PMO and Pip6e-PMO treated *mdx* animals and compared with age- and sex-matched wild-type and untreated *mdx* controls. DystromiR levels were restored to a greater degree in Pip6a-PMO animals compared with Pip6e-PMO for all dystromiRs analysed (miR-1, miR-133a and miR-206) (Figure 1D). miR-1 was restored to wild-type levels after a single administration of Pip6a-PMO (*P* < 0.01). These results suggest a dose-dependent effect whereby greater dystrophin rescue leads to greater restoration of circulating dystromiRs towards wild-type levels.

### Identification of novel differentially expressed serum dystromiRs

**Figure 2.**
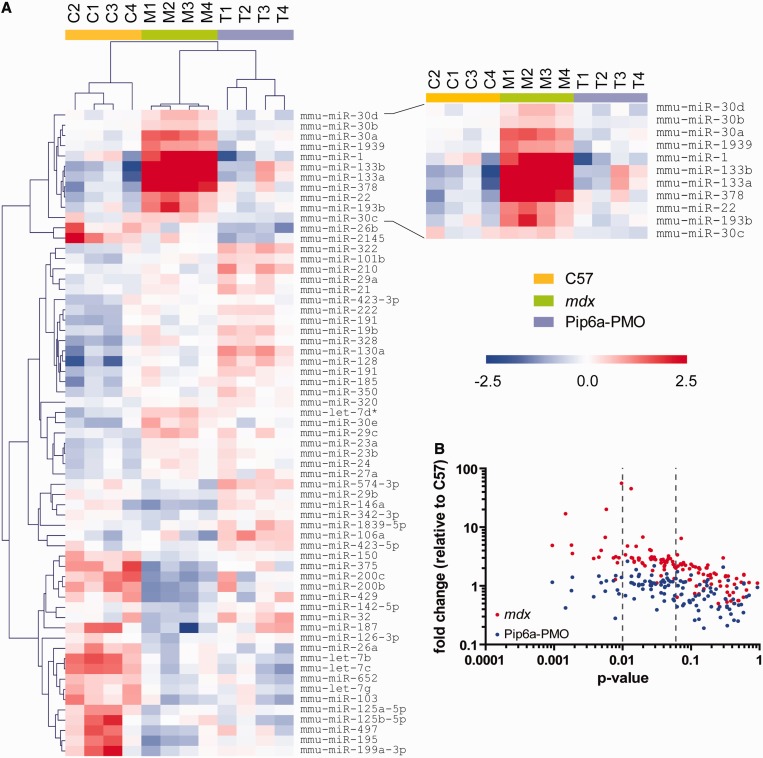
Identification of novel extracellular biomarkers by serum miRNA profiling. Serum from C57Bl/10, *mdx* and Pip6a-PMO-treated *mdx* mice was analysed by miRCURY LNA SYBR Green miRNA RT-qPCR array. (**A**) Heatmap of relative expression values for all miRNAs detected in all samples. Red indicates higher abundance, and blue indicates lower abundance. Scale bar values represent ΔΔCt values. The heatmap was produced by median centering expression values for each miRNA and then performing hierarchical clustering. (**B**) Plot of *P*-value versus fold change for miRNAs detected in all samples.

To identify novel differentially expressed serum dystromiRs, miRNA expression profiling was performed by miRCURY LNA SYBR green RT-qPCR array. The 12-week-old male *mdx* animals (*n* = 4) were treated with a single intravenous injection of Pip6a-PMO (12.5 mg/kg) and sacrificed 2 weeks later. Serum samples from these mice were compared with age- and sex-matched C57Bl/10 (wild-type) and untreated *mdx* serum. In all, 123 miRNAs were detected in all samples. The number of miRNAs detected in each sample varied from 168 to 258. Experimental groups clustered as expected when analysed by hierarchical clustering (Figure 2A) or principal component analysis (Supplementary Figure S1): treated samples clustered with the C57Bl/10 wild-type controls and away from the *mdx* samples. Fifteen miRNAs were significantly changed at the *P* < 0.01 level, and a further 42 miRNAs were significantly changed at the *P* < 0.05 level (one-way ANOVA). The abundance of all of these 57 miRNAs increased in *mdx* serum and was restored in the Pip6a-PMO-treated samples, suggesting a profound restoration of the circulating miRNA profile following treatment (Figure 2B). Aside from the established dystromiRs, the four most differentially abundant miRNAs were miR-22, miR-30a, miR-193b and miR-378 (Figure 2A). These candidate miRNAs were selected for further evaluation as putative biomarkers.

### Serum dystromiR abundance changes dynamically over time and following treatment

**Figure 3.**
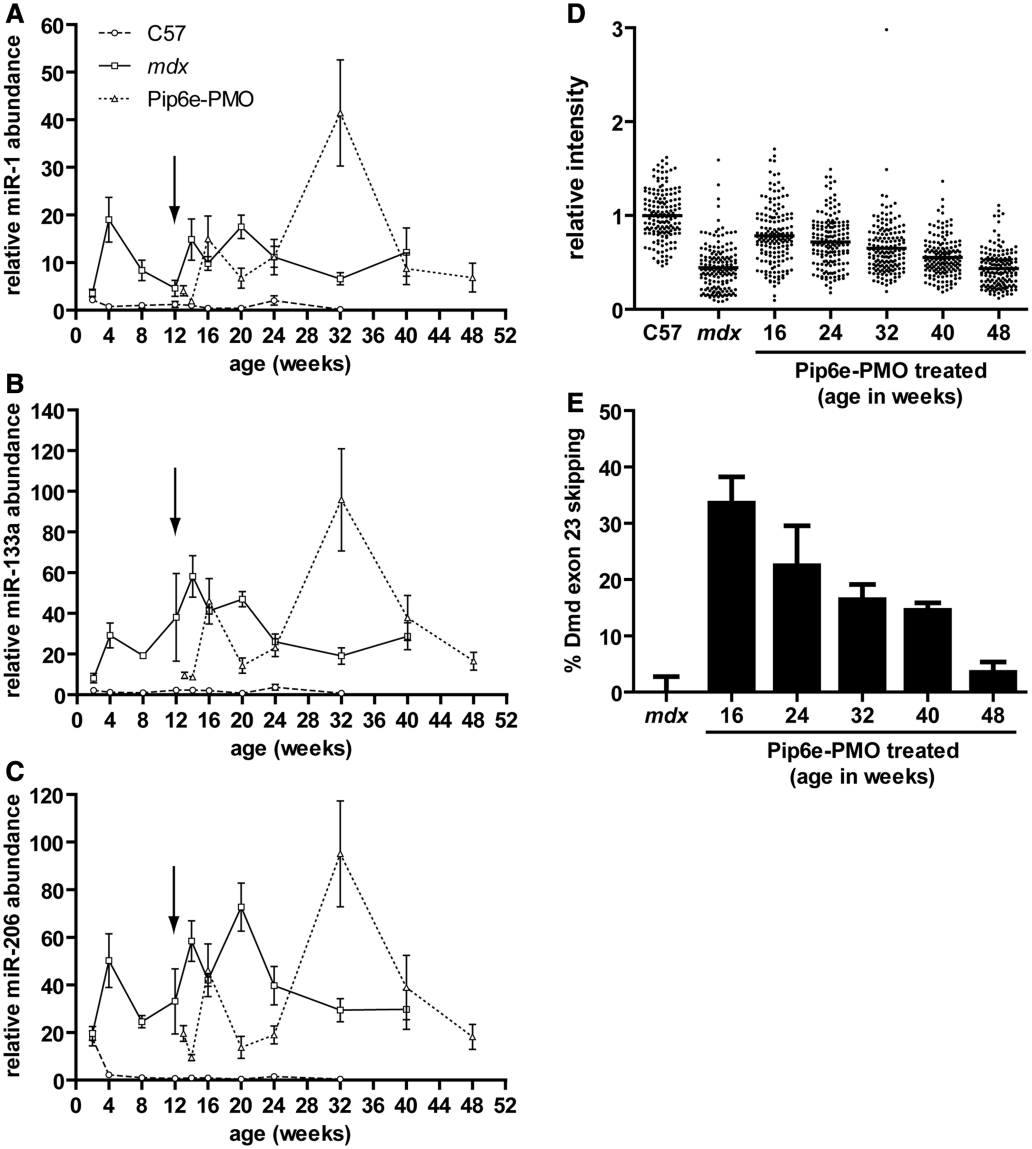
Time course of serum dystromiR abundance. Male C57Bl/10, *mdx* and Pip6e-PMO-treated *mdx* mice were sacrificed at various ages, and serum miRNA levels were determined by small RNA TaqMan RT-qPCR. (**A**) miR-1, (**B**) miR-133a and (**C**) miR-206 abundance was normalized to an external spike-in control. All miRNA expression data were normalized to the mean of the 8-week-old C57Bl/10 group. Arrow indicates time of injection (single intravenous 12.5 mg/kg dose Pip6e-PMO). The time course of exon skipping and dystrophin restoration was also determined in the TA of the treated *mdx* samples at various time points by (**D**) quantitative immunofluorescence and (**E**) RT-qPCR, respectively. Values are mean ± SEM, *n* = 3–8.

To investigate how serum miRNA levels change during disease progression or in response to dystrophin restoration C57Bl/10, *mdx* and *mdx* mice treated with Pip6e-PMO were sacrificed at various ages (between 2 and 48 weeks old) and serum miRNAs analysed by RT-qPCR (Figure 3A–C). Pip6e-PMO treated *mdx* mice were administered with a single 12.5 mg/kg intravenous injection at 12 weeks of age and TA, diaphragm and heart muscles subsequently dissected and dystrophin restoration determined by quantitative immunofluorescence (Figure 3D) and RT-qPCR (Figure 3E) at various time points.

In wild-type C57Bl/10 animals, serum dystromiR abundance was generally low (i.e. Ct values ∼35) and remained stable over the range of time points measured. One important exception was miR-206 which, at the 2-week time point, was ∼20-fold higher than at any other age investigated (Figure 3C). This observation illustrates that serum miRNAs can be elevated in the absence of muscle pathology.

In *mdx* animals, dystromiR levels were unchanged relative to C57Bl/10 controls at the 2-week time point. At all later time points measured, serum dystromiRs were elevated in *mdx* sera relative to age-matched C57Bl/10 controls (miR-1 range: 5.5–42-fold, miR-133a range: 19–58-fold and miR-206 range: 22–154-fold). The pattern of dystromiR abundance in *mdx* animals changed dynamically over time with all three dystromiRs exhibiting similar patterns of differential serum abundance. Local serum dystromiR maxima were observed at 4, 16 and 24 weeks and a local minimum observed at 12 weeks of age. Serum dystromiR levels remained relatively stable after the 24-week time point (although elevated relative to C57Bl/10 controls).

Following a single intravenous 12.5 mg/kg injection of Pip6e-PMO, serum dystromiR abundance was initially restored relative to wild-type animals (Figure 3A). Restoration could be detected as early as 1 week after injection, and levels remained low until 8 weeks after injection. By 12 weeks post-injection, miRNA levels in Pip6e-PMO treated mice were indistinguishable from untreated *mdx*. Following this, a spike in dystromiR abundance was observed at 32 weeks of age and then the dystromiRs returned to levels similar to the *mdx* control at 40 and 48 weeks of age.

In contrast, dystrophin protein recovery (Figure 3D) and the percentage of skipped Dmd transcripts (Figure 3E) in the TA muscle steadily declined over time following treatment, indicating that the pattern of serum dystromiR abundance is not a direct function of dystrophin expression levels.

To assess their suitability as disease biomarkers, the novel miRNAs identified by RT-qPCR array were also measured in the time course serum RNA samples (Supplementary Figure S2). Fluctuations in the abundance of these miRNAs broadly matched miR-1, miR-133a and miR-206 levels in Pip6e-PMO-treated mice, although the baseline levels in the C57Bl/10 and untreated *mdx* mice showed greater variation. Although these miRNAs were generally elevated in the *mdx* samples, for miR-22, miR-30a and miR-193b, the serum miRNA levels dropped below C57Bl/10 levels at the 32-week time point (Supplementary Figure S2A–C). In the case of miR-378, serum miRNA levels were elevated relative to C57Bl/10 controls at every time point measured (Supplementary Figure S2D). miR-378 also showed the greatest magnitude changes in relative serum levels (range: 1.4–12-fold) of the set of novel candidate miRNAs (although these fold changes are considerably less than those observed with the dystromiRs described earlier in the text).

### Serum dystromiR abundance correlates with markers of muscle regeneration

**Figure 4.**
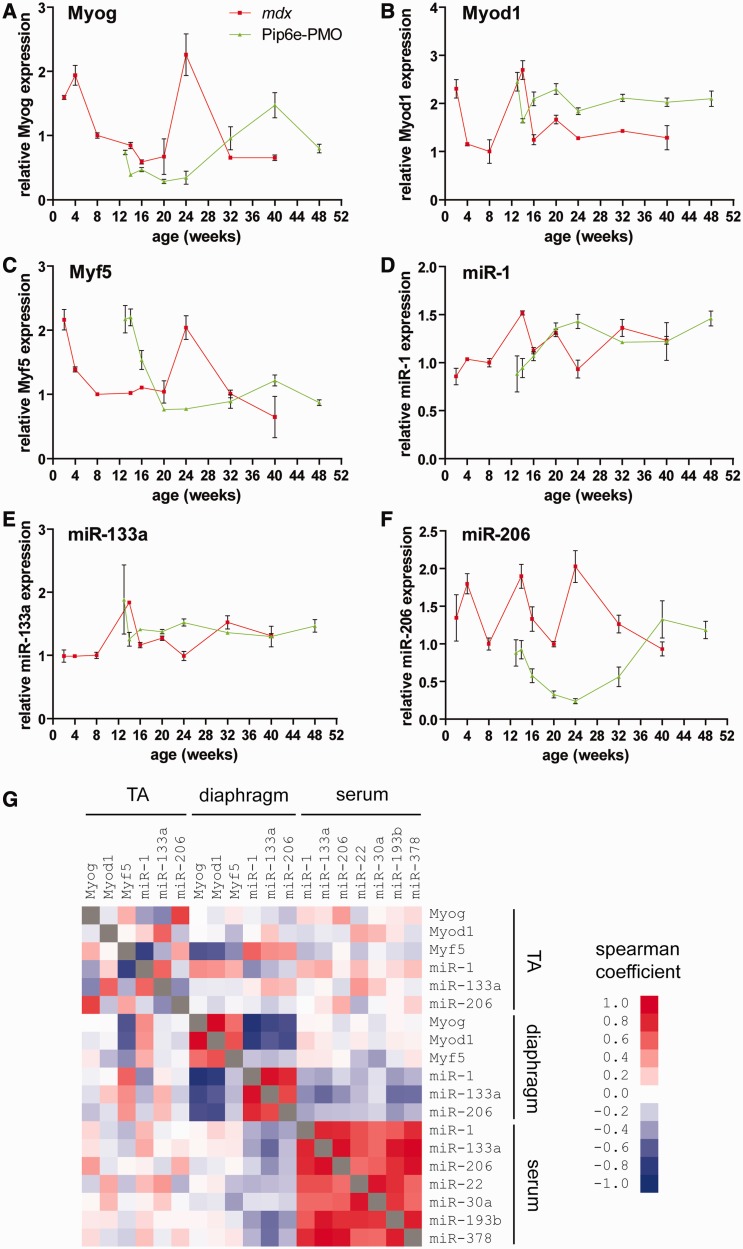
Serum dystromiR abundance correlates with expression of regeneration factors in the TA. TA muscles from *mdx* and Pip6e-PMO-treated *mdx* mice were harvested at various time points, and the expression of the myogenic transcription factors (**A**) Myog, (**B**) Myod1 and (**C**) Myf5 determined by RT-qPCR normalized to the geometric mean of the housekeeping genes Actb, Tbp, Rplp0 and Rpl10. Similarly, the same tissues were analysed for expression of (**D**) miR-1, (**E**) miR-133a and (**F**) miR-206 by small RNA TaqMan RT-qPCR normalized to miR-16 expression. All values are mean + SEM, *n* = 3–4. (**G**) Correlation matrix comparing the pattern of expression across all comparable time points of all myogenic transcription factors, dystromiRs and novel miRNA biomarkers between in the TA, diaphragm and in serum. Scale bars indicate Spearman correlation coefficients. Red indicates a positive correlation, blue indicates a negative correlation and white indicates no correlation.

Given that both the previously described dystromiRs and the novel candidate miRNA biomarkers identified here showed complex patterns of serum abundance over time, which did not follow the pattern of dystrophin restoration, we reasoned that these miRNAs might reflect underlying pathophysiological processes occurring in muscle. As the dystromiRs of interest have established roles in the control of muscle development, we measured the expression of the myogenic transcription factors myogenin (Myog), Myod1 and Myf5 and the dystromiRs themselves by RT-qPCR in the TA (Figure 4A–F) and diaphragm (Supplementary Figure S3A–F) muscles, harvested from the same mice used in the time course study. To investigate the relationship between patterns of expression in the tissues and in serum, correlation analyses were performed between mRNAs/miRNAs over all comparable time points (Figure 4G, Supplementary Table S1).

In the TA, expression of Myog, Myf5 and miR-206 was positively correlated. In *mdx* TA, expression levels of these factors were high at early time points, dropping at ∼16 weeks of age, rising to a second maximum at age 24 weeks and then finally dropping at 40 weeks. Treatment with Pip6e-PMO resulted in a decrease in expression that reached a minimum at 24 weeks before peaking again at the 40-week time point. These results suggest that restoration of dystrophin leads to a reduction in the pathophysiological cycles of degeneration and regeneration in the TA (i.e. stabilization of muscle turnover). As this treatment is only a single dose, the restored dystrophin protein is eventually degraded. This leads to recurrence of dystrophic pathology, thus accounting for the increases in Myog, Myf5 and miR-206 expression observed at later time points. In contrast expression of Myod1, miR-1 and miR-133a was relatively stable over the period measured, with only small changes observed longitudinally between time points and small fold change differences between *mdx* and Pip6e-PMO treated *mdx* mice at age-matched time points.

The expression patterns in the diaphragm were different to those observed in the TA (Supplementary Figure S3). In the diaphragm, the myogenic transcription factors all followed a similar pattern (and showed strong positive correlations with one another), whereby expression was relatively stable in the *mdx* samples with the exception of the 14-week time point, where a drop in expression was observed. In the Pip6e-PMO-treated animals, a similar pattern was observed, although with a 2-week lag following the time of injection, suggesting a short period of delayed pathology. Similarly, all three dystromiRs showed similar patterns of expression and strongly positive correlation coefficients. DystromiR expression changed little in the *mdx* diaphragm, whereas Pip6e-PMO treated tissues showed a 1.5–2.5-fold increase in expression at the 13–16-week time points before returning to levels seen in the *mdx* mice. In contrast with the TA, the myogenic transcription factors were positively correlated with each other but inversely correlated with expression of the dystromiRs. These observations underline the fundamental difference in the way dystrophic molecular pathology manifests in the diaphragm as opposed to other skeletal muscles.

Comparison of the expression of myogenic transcription factors and dystromiRs in the TA with the serum dystromiR abundance revealed some important similarities. The patterns of tissue expression for Myog (Figure 4A), Myf5 (Figure 4C) and miR-206 (Figure 4F) resemble the patterns of serum dystromiR abundance (Figure 3A–C), and this was reflected in the correlation analysis (although correlation coefficients were weaker than for within tissue comparisons) (Figure 4G). Interestingly, although similar peaks were observed in dystromiR/myogenic factor expression between the TA and serum, these occurred at different times (being offset by 1–2 months). These results suggest that the spikes in serum miRNA levels precede the corresponding elevation of dystromiRs/myogenic factors in the TA. Alternatively, the relative contribution of the TA to the totality of circulating dystromiRs may be small. It has been reported previously that dystrophic pathology is asynchronous between different muscles (25). It is therefore possible that other muscles may be undergoing pathological processes that more closely correlate with the pattern of serum miRNA abundance.

### Serum dystromiRs are released in response to experimentally-induced muscle injury

**Figure 5.**
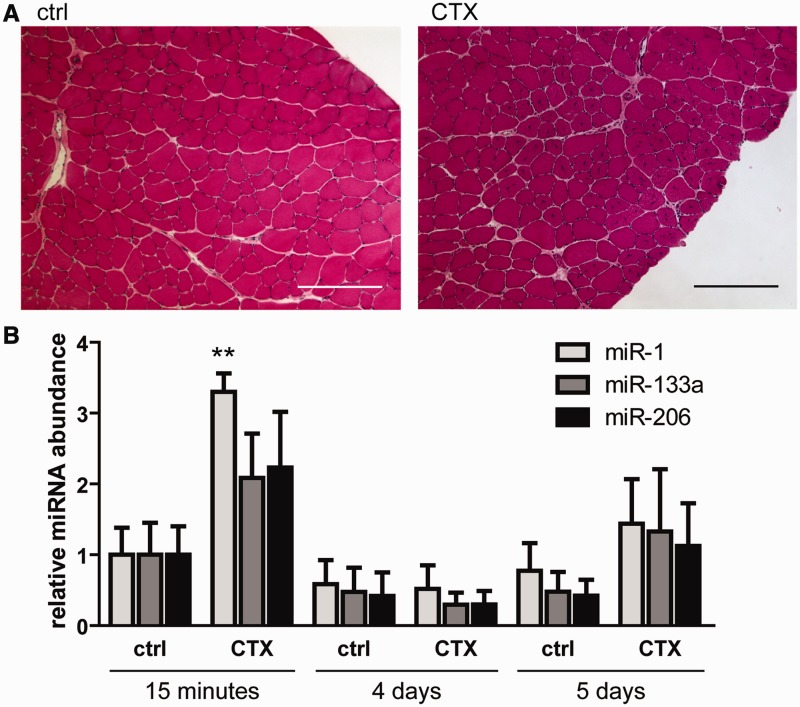
CTX injury induces release of serum dystromiRs. The 8-week-old male C57Bl/10 (ctrl) mice were injected with CTX in the TA and serum harvested at 15 min, 4 days and 5 days post-injection. (**A**) CTX injury was confirmed by Hematoxylin and Eosin staining of transverse sections through the mid-belly of the TA muscles. CNFs were abundant in CTX-treated muscle indicating widespread regeneration. (**B**) Serum dystromiR levels were determined by small RNA TaqMan RT-qPCR normalized to an external spike-in control oligonucleotide. Values are mean + SEM, *n* = 4 mice, **P < 0.01. Scale bars represent 100 μm.

To further test the hypothesis that serum dystromiRs reflect the regenerative status of muscle we used a localized acute muscle injury model. The TA muscles of 8-week-old male C57Bl/10 mice were injected with CTX, animals were harvested 14 days after injection and TA muscles were dissected. The injection of CTX induced local muscle regeneration as determined by histological staining. CNFs, a marker of regeneration, were counted in a series of haematoxylin and eosin-stained sections. In CTX-treated muscles, the mean CNF count was 100 (SD = 46), indicating that muscle injury had been successfully induced (Figure 5A). No CNFs were detected in the control tissues. To investigate extracellular miRNA release, serum was collected via the tail vein at 15 min, 4 days and 5 days post-injection and serum miRNA levels determined by small RNA TaqMan RT-qPCR compared with untreated controls (Figure 5B). An increase in all three serum dystromiR levels was detected in the CTX injected animals at 15 min, and a trend towards an increase was observed at 5 days post-injection (although this did not reach statistical significance). The fold changes in serum dystromiR abundance following CTX injection were much lower than those observed in *mdx* mice. This is unsurprising given that, in the *mdx* mouse, extracellular dystromiRs likely originate from the entire musculature, whereas in the injury model, the dystromiRs are derived from the TA muscles only. These results suggest that serum dystromiRs are released into the serum during acute muscle degeneration (15 min time point) and possibly also during the regenerative response (5-day time point) (26).

### Serum dystromiRs are primarily non-vesicular and protein bound

**Figure 6.**
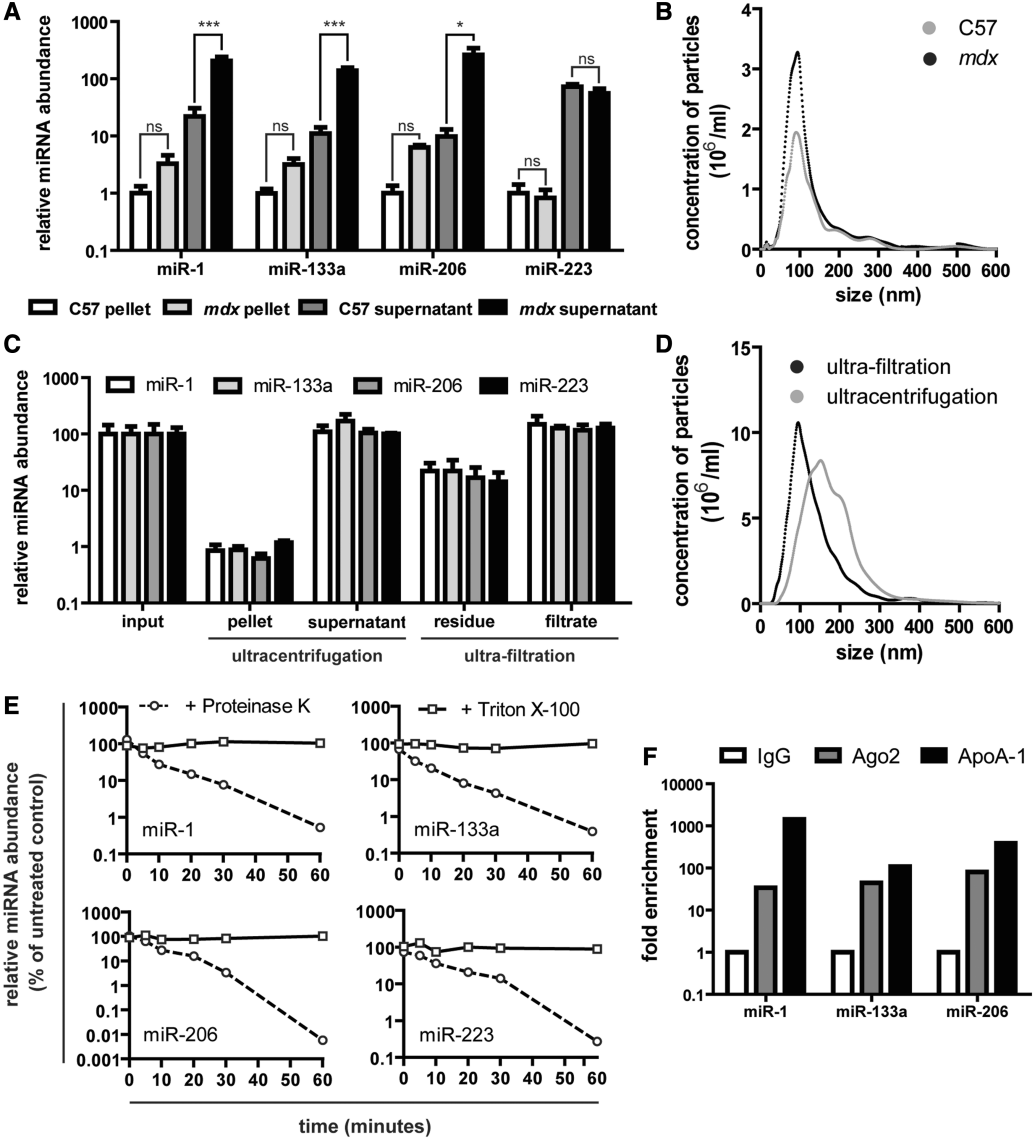
Serum dystromiRs are primarily non-vesicular and are protected in protein complexes. Serum from C57Bl/10 and *mdx* mice was separated by centrifugation at 30 000 *g* for 1 h, and the pellet and supernatant fractions were analysed by (**A**) small RNA TaqMan RT-qPCR and (**B**) NTA. miR-223 was analysed, in addition to the dystromiRs miR-1, miR-133a and miR-206, as this miRNA was not expected to change between C57Bl/10 and *mdx* samples. *mdx* serum was separated by ultracentrifugation at 100 000 *g* for 1 h or ultra-filtration using 1 MDa filters, and the different fractions were analysed by (**C**) small RNA TaqMan RT-qPCR and (**D**) NTA. Samples are mean + SEM, *n* = 3, *P < 0.05, ***P < 0.001, ‘ns’ not significant. (**E**) Serum samples were treated with either 1 mg/ml Proteinase K at 55°C or 1% Triton X-100, aliquots removed at various intervals and analysed by small RNA TaqMan RT-qPCR. miRNA levels were compared with untreated controls. (**F**) RNA immunoprecipitation analysis of *mdx* serum samples. Protein-miRNA complexes were precipitated with antibodies against Argonaute-2 (Ago2) and Apolipoprotein A-1 (ApoA-1) and miRNA levels measured in the immunoprecipitates. Enrichment was determined relative to a negative control IgG antibody.

The extra-cellular environment is rich in RNases. A recent study showed that exogenous RNAs added to serum and plasma samples were rapidly degraded (∼1% remaining after 15 s exposure) (27). Despite these observations, miRNAs are readily detectable and highly stable in serum and plasma (10,11). Extracellular miRNAs are known to be released in lipid membrane-bound vesicles [e.g. microvesicles, exosomes, (28,29) apoptotic bodies (30)], protein complexes [e.g. Argonaute-2 (31), Argonaute-1 (32), nucleophosmin-1 (33)] or in lipoprotein complexes (e.g. high-density lipoprotein) (34). The sequestration of extracellular miRNAs in lipid vesicles or in complex with (lipo)proteins provides an explanation for their insensitivity to RNase-mediated degradation. We have previously postulated that dystromiRs reside within endogenous lipid vesicles (i.e. exosomes or microvesicles), thus accounting for their stability in serum and providing a biologically plausible mechanism for their activity (12). To test this hypothesis, we purified EVs from *mdx* and control serum by centrifugation. The relative levels of dystromiRs in the pellet (vesicular) and supernatant (non-vesicular) fractions were determined by RT-qPCR. miR-223 was also analysed, as this miRNA has been used previously by our group and others as an endogenous normalization control, and its abundance was therefore not expected to change significantly (12,13,15) (Figure 6A). DystromiRs were enriched in the supernatant fraction, suggesting that the majority (∼99%) of serum dystromirs are non-vesicular.

Centrifuged serum samples were characterized by NTA, which demonstrated a well-defined EV population in the pelleted samples (Figure 6B). The modal size of the nanoparticles was ∼80 nm, consistent with the properties of exosomes and similar between the *mdx* and control sera (Supplementary Figure S4A). A small but non-significant increase in the number of particles was observed in the *mdx* samples relative to the control samples (Supplementary Figure S4B). The majority of serum RNA was found in the supernatants and not in the microvesicle pellet as measured by RiboGreen assay (Supplementary Figure S4C). Total RNA yields were generally higher in both the *mdx* pellets and supernatants relative to the C57Bl/10 control, although the differences were not statistically significant.

To more rigorously investigate vesicular miRNA content, *mdx* serum was separated by ultracentrifugation at 100 000 *g* for 1 h or ultra-filtration with 1 MDa filters. The different fractions were analysed by RT-qPCR (Figure 6C) and NTA (Figure 6D). Approximately 1% of the serum miRNAs were pelleted by ultracentrifugation, whereas between 10 and 20% of miRNAs remained in the filters. The results were similar between the dystromirs (miR-1, miR-133a and miR-206) and the control miRNA (miR-223). These results are consistent with the majority of serum miRNAs being non-vesicular. Serum samples used in these analyses were frozen after collection. To rule out the possibility that the freezing process was disrupting vesicles and releasing their miRNA contents, the same analysis was performed on fresh *mdx* serum with similar results (Supplementary Figure S4D), suggesting that freeze-thawing has little or no effect on the stability of vesicular miRNAs.

Previous studies have shown that extracellular miRNAs can be destabilized by treatment with Proteinase K (35) or detergents (36,37) that expose miRNAs to serum RNases by disrupting protein complexes or the lipid membranes of EVs, respectively. To investigate the nuclease stability of the dystromiRs of interest, serum samples were treated with either Proteinase K (at 55°C) or Triton X-100 for 1 h. Aliquots of serum were removed at various time points and TRIzol LS added immediately to prevent further RNA degradation. miRNA abundance was assessed by RT-qPCR, normalized to an external spike-in control miRNA and compared with untreated serum samples. A rapid decrease in miRNA abundance was observed in the Proteinase K-treated serum samples (Figure 6E). Interestingly, even incubation at 55°C for 1 h had little effect on the levels of miRNAs in the untreated samples underlining the highly stable nature of miRNAs in serum. Conversely, treatment with Triton X-100 did not result in dystromiR destabilization indicating that these miRNAs are protected from RNase-mediated degradation by association with proteins rather than by encapsulation in EVs. RNA immunoprecipitation was performed to determine the protein-binding partners of the dystromiRs of interest. All the miRNAs assayed immunoprecipitated with antibodies against Argonaute-2 and Apolipoprotein A-1 (Figure 6F), suggesting that the formation of complexes with these proteins may account for their serum nuclease stability.

## DISCUSSION

Although extracellular miRNAs have been identified in numerous studies as potential biomarkers, their biological and clinical significance remain largely unknown. Here, we report an in-depth analysis of circulating miRNA biomarkers in a mouse model of the degenerative muscle disease, DMD. The abundance of serum miRNAs changes dynamically over time, mirroring the progression of muscle pathology in the *mdx* mouse and following the expression pattern of regenerative factors in skeletal muscle. Serum dystromiRs can thus be considered biomarkers of muscle turnover.

This study adds to the growing literature supporting the use of serum miRNAs as biomarkers for DMD. Serum miRNA profiling showed a profound restoration in the serum miRNA signature after treatment with exon skipping therapy (Figures 1D and 2A and B). Importantly, we have shown that greater dystrophin rescue is associated with greater serum miRNA restoration. Similarly, Cacchiarelli *et al.* (13) showed that patients with the less severe dystrophinopathy, Becker muscular dystrophy, have intermediate levels of serum miRNA levels relative to DMD patients and healthy controls. Serum dystromiRs were consistently elevated in *mdx* mice, suggesting that these are suitable biomarkers for distinguishing affected and unaffected patients or animal models. Following a single intravenous treatment with Pip6e-PMO, circulating dystromiRs were restored for several months (Figure 3A–C). Interestingly, dystrophin expression returned to the same levels as *mdx* mice 20 weeks after injection (age 32 weeks), whereas serum dystromiRs were at their most abundant, indicating that serum miRNAs levels are not simply a function of dystrophin protein expression (Figure 3D and E). Additionally, we have identified four novel biomarkers (miR-22, miR-30a, miR-193b, miR-378) by serum miRNA profiling and validated these with individual RT-qPCR assays. These novel candidate miRNAs behave similarly over time, and in response to dystrophin restoration, to the previously identified dystromiRs, suggesting they be useful disease biomarkers. Some of these miRNAs have already been implicated in muscle or cardiac-specific processes (38–40), and therefore their involvement in DMD pathophysiology warrants further investigation.

It is highly likely that the dystromiRs discussed in this study originate from muscle for a number of reasons. These miRNAs have been widely described as ‘muscle-specific’. miR-1 and miR-133a are primarily expressed in skeletal and cardiac muscle, and miR-206 is restricted to skeletal muscle (41) and the role of these ‘myomiRs’ in muscle development and regeneration is already well established (41,42), suggesting that these extracellular dystromiRs are unlikely to originate in non-muscle tissues. Moreover, we provide evidence that dystromiRs are released into the circulation following acute experimentally induced muscle injury, which is further evidence of their muscle origins.

The dystromiRs miR-1, miR-133 and miR-206 are present at low levels in myogenic precursor cells, are upregulated during myogenic differentiation and can be considered markers of adopting a muscle lineage (8,41). It is therefore unlikely that extracellular dystromiRs originate from the satellite stem cell pool. We have previously reported that miR-206 is upregulated in the TA of 8-week-old *mdx* relative to wild-type controls, whereas the expression of miR-1 and miR-133a was unchanged (12). This difference in expression between these dystromiRs is recapitulated in the results presented here, whereby miR-206 exhibits a dynamic pattern of expression over time in muscle, whereas miR-1 and miR-133a are relatively stable (Figure 4D–F). Elevated levels of miR-206 in dystrophic muscle can be explained by an increase in muscle regeneration, as this miRNA is predominantly expressed in newly regenerating fibres (43) and is elevated during the regenerative phase following CTX injury (44). Consequently, the asymmetry in miRNA expression between serum and muscle suggests that regenerating fibres contribute substantially to the release of dystromiRs into the circulation.

Despite the absence of dystrophin protein, the dystrophic pathology of the *mdx* mouse is less severe than in DMD patients and follows a differing natural history. At age 3–4 weeks, the *mdx* mouse experiences a degenerative phase characterized by widespread myofibre necrosis, central nucleation of fibres indicative of regeneration, infiltration of immune cells (e.g. macrophages) and elevated serum creatine kinase (45,46). By 12 weeks of age, the necrosis is largely replaced with regeneration, and there is a reduction in immune infiltrate (47). By analysing the levels of serum miRNAs for a ∼12-month period, we have shown that serum dystromiR abundance changes dynamically over time, and that the pattern of dystromiR abundance mirrors the pathological features observed in *mdx* muscle (Figure 3A–C). There is a peak of serum dystromiR levels at age 4 weeks consistent with the early crisis period of extensive muscle damage and a second peak starting at the 12–14 week period consistent with the regenerative phase described earlier in the text. Exon skipping therapy restores the levels of serum dystromiRs for ∼1 month and effectively delays the peaks in dystromiR abundance observed in *mdx* serum. Interestingly, at 32 weeks of age, all three dystromiRs reached a global maximum in the treated samples. A possible explanation for this is that synchronization of pathology occurs between muscles following treatment such that multiple muscles are undergoing regeneration simultaneously at this later time point.

Further investigation of the TA muscles harvested from the time course study revealed an interesting similarity between the expression patterns of the myogenic transcription factors myogenin and Myf5 (Figure 4A and C) and serum dystromiR levels (Figure 3A–C). Importantly, this result suggests that serum miRNA biomarkers can provide information about the physiological state of muscle without the need for an invasive biopsy. Similarly, a small increase in serum dystromiRs was observed in the serum of animals injected with CTX 5 days post-injection, a time point at which muscle regeneration is known to be taking place (26,44) (Figure 5). Taken together, these data suggest that the levels of serum dystromiRs reflect the regenerative status of the musculature.

Several lines of evidence are suggestive of selective release of miRNAs following muscle injury. First, we have previously reported that the pattern of muscle miRNA expression does not match the pattern of miRNA abundance in serum (12). Second, we have observed that miRNAs that are highly enriched in skeletal muscle (let-7a/b/c/f/g) (48) were either not detected in mouse serum samples or did not change their relative serum abundances between C57Bl/10 and *mdx* samples (Supplementary Data File S1). Furthermore, levels of miR-206 were elevated in wild-type animals at 2 weeks of age, demonstrating that changes in serum miRNA abundance can occur independently of muscle damage (possibly as a result of muscle growth and development in young mice). It is possible that all three miRNAs originate from regenerating fibres, although the high levels of miR-1 and miR-133 expression in mature muscle mean that this is not trivial to demonstrate. However, release of all three dystromiRs immediately after CTX injury suggests that they can also reach the circulation following muscle degeneration.

Selective release of miRNAs by exocytosis would provide a plausible explanation for the abundance of muscle-enriched specific miRNAs in *mdx* and DMD patient serum. However, the results of this study show that the vast majority of serum miRNAs are non-vesicular at time of detection (Figure 6). This would imply either that miRNAs are initially released in vesicles, which are subsequently degraded by serum phospholipases, or that there are specific protein-miRNA complex export pathways independent of vesicle release. Whether circulating miRNAs are capable of being taken up by recipient cells and influencing gene expression (thus constituting a signalling function) remains an open question. Several reports have shown that circulating miRNAs are taken up by target cells where they are biologically active (28,29,49), and exosomes have recently been shown to deliver exogenous siRNA cargoes (50). Interestingly, local injection of muscle-specific miRNAs has been shown to enhance muscle regeneration in injured rat muscle (51), and forced expression of miR-1 in HeLa (non-muscle) cells alters their transcriptional profile to become more muscle-like (52). Taken together, these studies suggest that extracellular miRNAs might be able to induce phenotypic changes in recipient cells.

We propose a model whereby serum dystromiRs are released by regenerating and/or degenerating mature fibres. The miRNAs are initially contained within EVs and act to signal to neighbouring satellite cells to stimulate a regenerative response. This would constitute a paracrine signal from damaged muscle instructing the muscle stem cell population to activate to maintain muscle homeostasis. miRNAs are over-released such that a proportion fail to reach their intended target cells. These miRNAs enter the circulation where their vesicular carriers are gradually degraded. miRNAs remain nuclease stable owing to their association with proteins such as Argonaute—2, or through the formation of complexes with lipoproteins.

In conclusion, serum dystromiRs can be considered biomarkers of muscle turnover (i.e. myofibre degeneration and regeneration). These miRNAs are differentially released over time from mature or regenerating fibres and are primarily non-vesicular at the time of detection. Given that serum dystromiRs are currently under investigation for disease monitoring in exon skipping clinical trials, we hope that the findings of this study will aid the clinical interpretation of the levels of these biomarkers. Similarly, the longitudinal analysis of dystromiR abundance presented here should be considered in the design of future patient trials to determine the optimal time points for taking measurements.

## SUPPLEMENTARY DATA

Supplementary Data are available at NAR Online.

## FUNDING

Association Française Contre les Myopathies [14784 to M.J.A.W.]; Muscular Dystrophy Campaign [RA1/836/1 to M.J.A.W.]; Wellcome Trust [091982 to M.J.A.W., 08421/Z/07/Z to J.E.M.]; Netherlands Organization for Scientific Research [825.11.032 to P.V.]. Funding for open access charge: Association Française Contre les Myopathies.

*Conflict of interest statement*. None declared.

## Supplementary Material

Supplementary Data
