# Supplementary material for: Extracellular microRNAs are dynamic non-vesicular biomarkers of muscle turnover
Source: Nucleic Acids Res. 2013 Aug 14;41(20):9500–13. doi: 10.1093/nar/gkt724 (PMC3814379; doi:10.1093/nar/gkt724)
Supplement: Supplementary Data [file supp_41_20_9500__index.html]

Extracellular microRNAs are dynamic non-vesicular biomarkers of muscle turnover — Extracellular microRNAs are dynamic non-vesicular biomarkers of muscle turnover — Supplementary Data 

# Extracellular microRNAs are dynamic non-vesicular biomarkers of muscle turnover

## Supplementary Data

files

**Files in this Data Supplement:**

- Supplementary Data - pdf file
- Supplementary Data - xls file
