# Supplementary material for: Extracellular microRNAs are dynamic non-vesicular biomarkers of muscle turnover
Source: Nucleic Acids Res. 2013 Aug 14;41(20):9500–13. doi: 10.1093/nar/gkt724 (PMC3814379; doi:10.1093/nar/gkt724)
Supplement: Supplementary Data [file supp_gkt724_nar-01470-y-2013-File008.pdf]

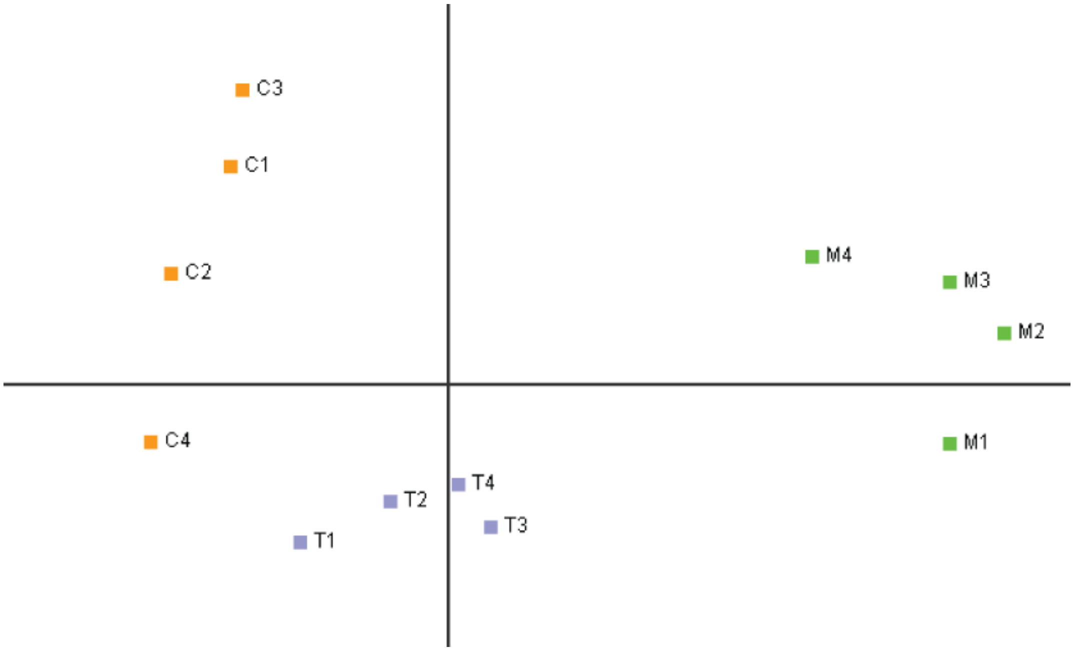


**Fig. S1**

**Principal component analysis of serum microRNA profile**

Principal component analysis was performed on ΔΔCt data from the miRCURY LNA SYBR Green RT-qPCR array for all miRNAs that were detected in all samples (123 assays). The first two components are shown representing a data reduction comprised of 82% of the data. Experimental groups cluster as expected. Samples are C57Bl/10 (C1-4), *mdx* (M1-4) and Pip6a-PMO treated *mdx* (T1-4). Treated samples cluster towards the C57Bl/10 control samples and away from the *mdx* samples.

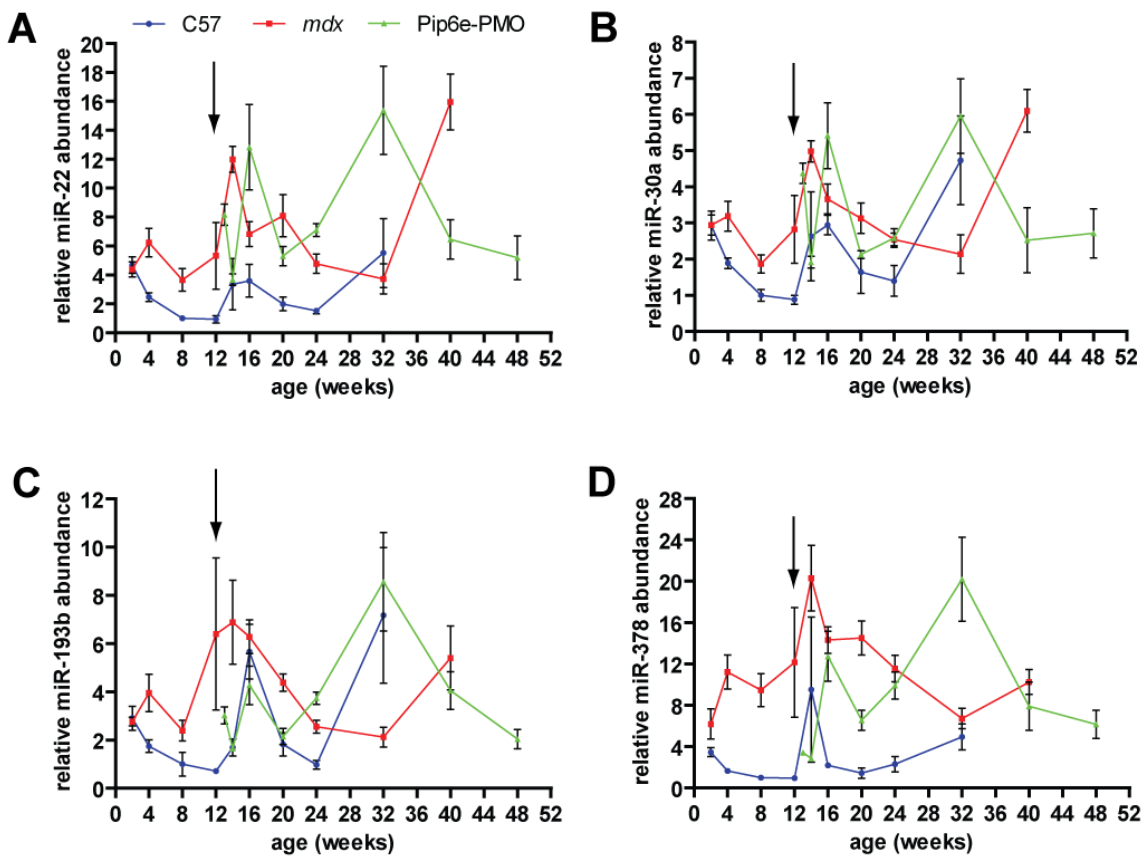


**Fig. S2**

**Time course of novel serum biomarker abundance**

Male C57Bl/10, *mdx* and Pip6e-PMO treated *mdx* mice were sacrificed at various ages and serum miRNA levels determined by small RNA TaqMan RT-qPCR. (A) miR-22, (B) miR-30a, (C) miR-193b and (D) miR-378 abundance was normalized to an external spike-in control. All miRNA expression data were normalized to the mean of the 8 week old C57Bl/10 group. Arrow indicates time of injection (single intravenous 12.5 mg/kg dose Pip6e-PMO).

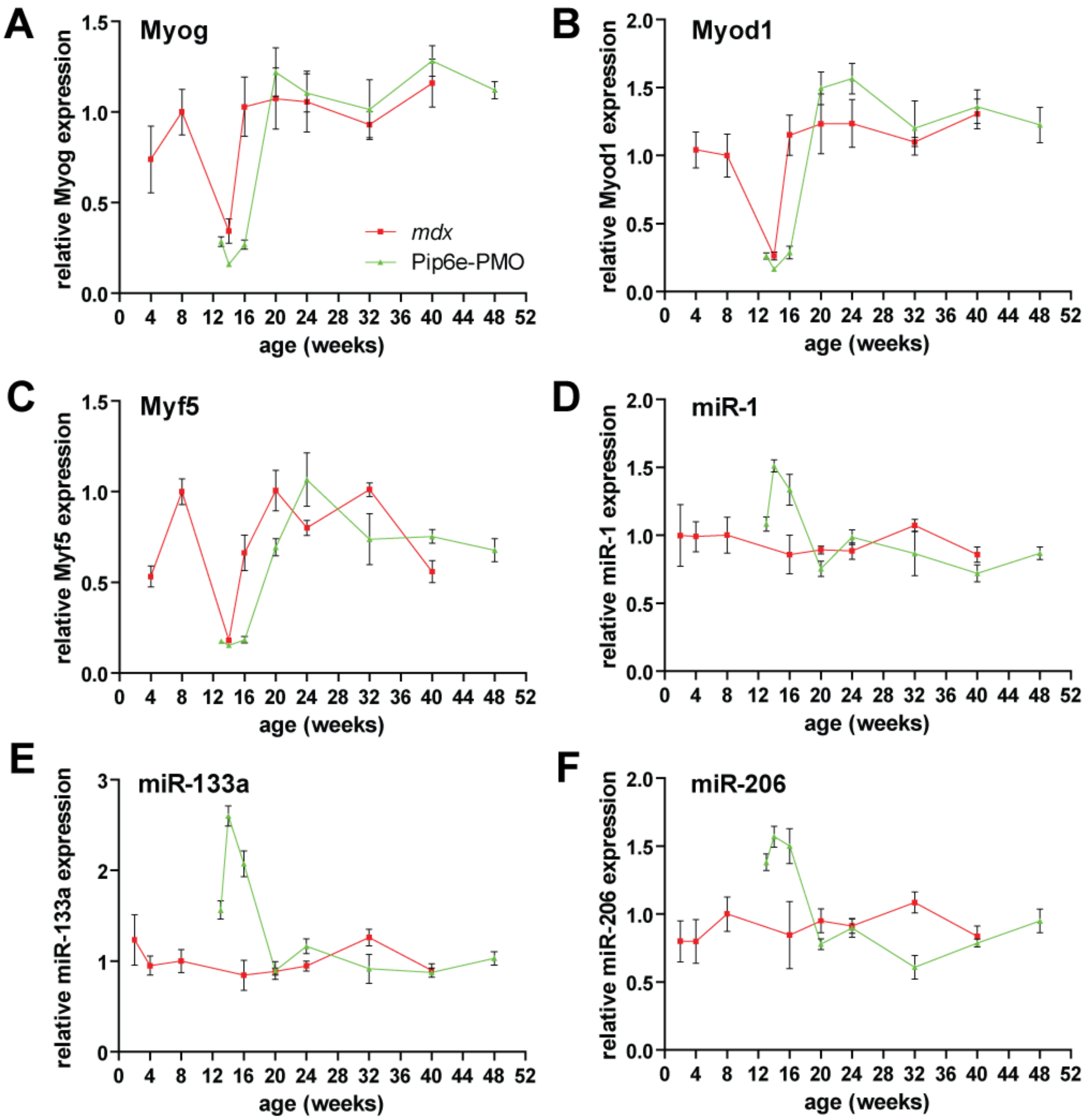
A
Myog
relative Myog expression
mdx
Pip6e-PMO
age (weeks)
B
Myod1
relative Myod1 expression
age (weeks)
C
Myf5
relative Myf5 expression
age (weeks)
D
miR-1
relative miR-1 expression
age (weeks)
E
miR-133a
relative miR-133a expression
age (weeks)
F
miR-206
relative miR-206 expression
age (weeks)

**Fig. S3**

**Expression of myogenic transcription factors and dystromiRs in *mdx* diaphragm**

Diaphragm muscles from *mdx* and Pip6-e-PMO treated *mdx* mice were harvested at various time points and the expression of the myogenic transcription factors (A) Myog, (B) Myod1 and (C) Myf5 determined by RT-qPCR normalized to the geometric mean of the housekeeping genes Actb, Tbp, Rplp0 and Rpl10. Similarly, the same tissues were analyzed for expression of (D) miR-1, (E) miR-133a and (F) miR-206 by small RNA TaqMan RT-qPCR normalized to miR-16 expression. All values are mean + SEM, n=3-4.

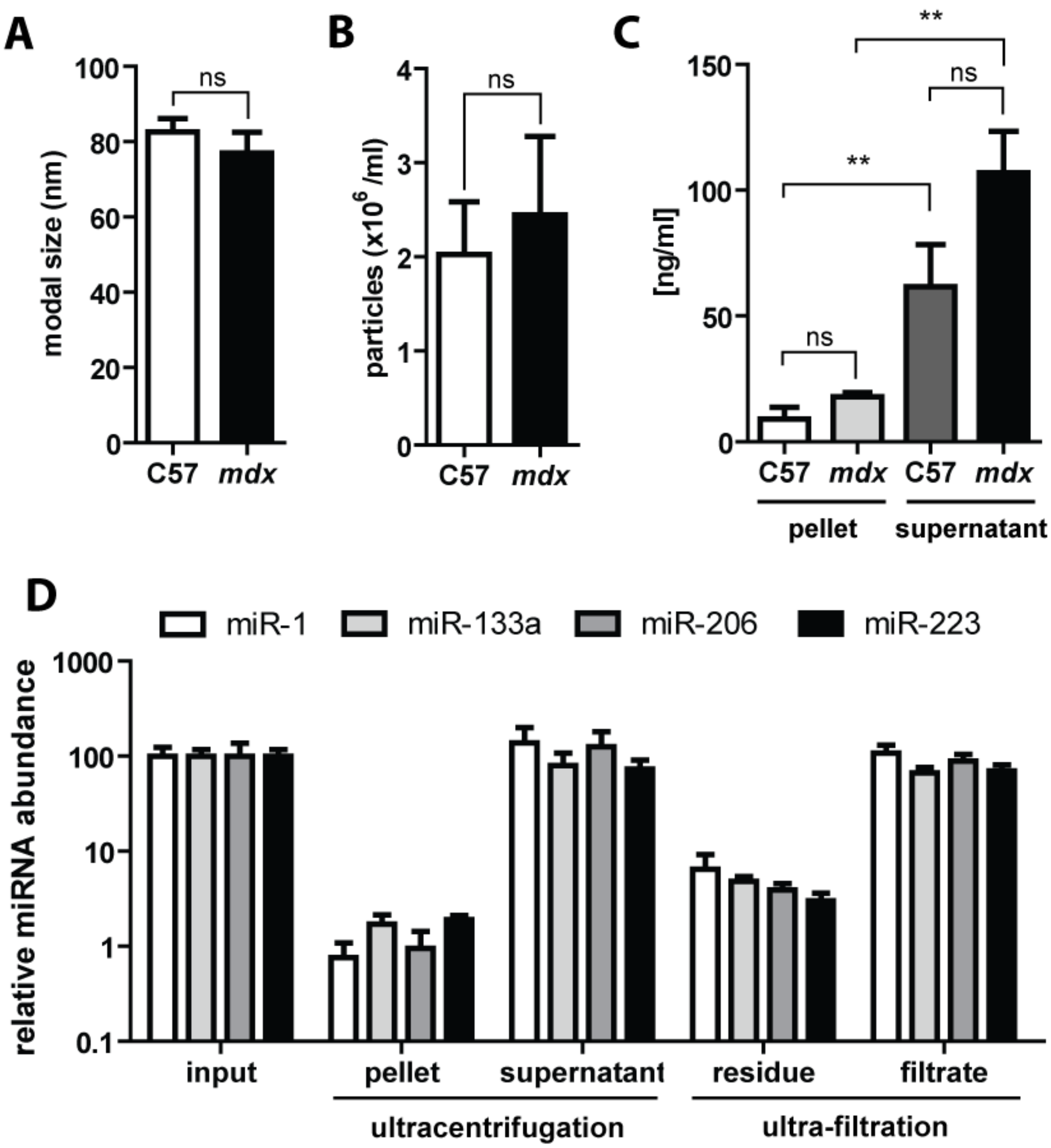
A
modal size (nm)
100
80
60
40
20
0
ns
C57
mdx
B
particles (x10^6 /ml)
4
3
2
1
0
ns
C57
mdx
C
[ng/ml]
150
100
50
0
**
ns
**
ns
C57
mdx
C57
mdx
pellet
supernatant
D
miR-1
miR-133a
miR-206
miR-223
relative miRNA abundance
1000
100
10
1
0.1
input
pellet
supernatant
residue
filtrate
ultracentrifugation
ultra-filtration

**Fig. S4**

**Characterization of extracellular vesicles from C57 and *mdx* serum**

C57Bl/10 and *mdx* serum separated by centrifugation at 30,000g for 1 hour and the modal size particle size (A) and particle counts (B) were determined for the resupended pellets by nanoparticle tracking analysis. (C) Total RNA content of the pellet and supernatant fractions was determined by RiboGreen assay. (D) Separately, male *mdx* mice (n=3) were sacrificed, serum harvested and samples analyzed before freezing. Serum was separated by ultracentrifugation (100,000g for 1 hour) or ultra-filtration (1 Mda filters) and RNA extracted from each fraction. miRNA abundance was measured by small RNA TaqMan RT-qPCR and normalized to an external spike-in control oligonucleotide and compared to an unseparated 'input' fraction. All values are mean + SEM, n=3. **$p<0.01$, 'ns' not significant.

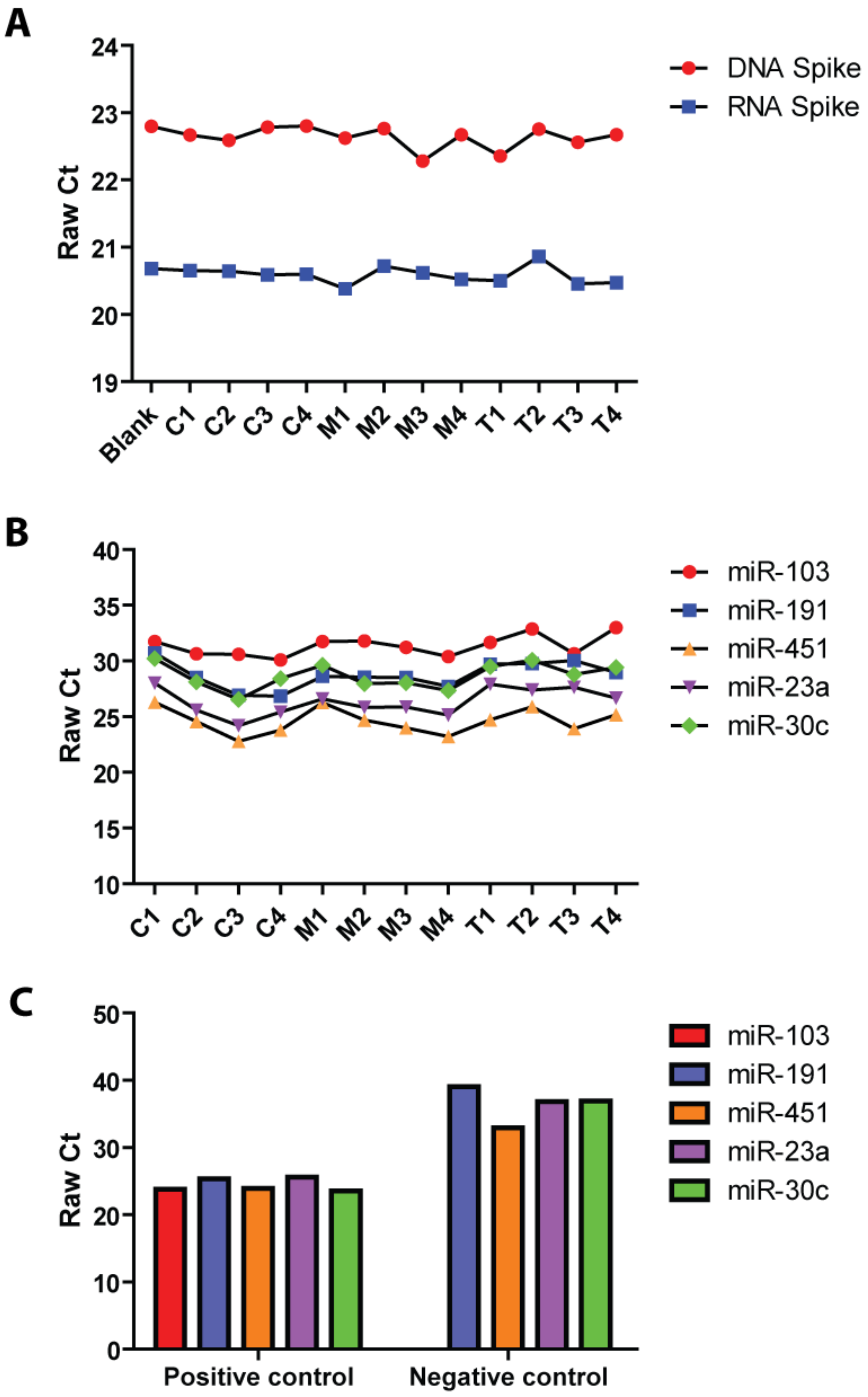
A
Raw Ct
24
23
22
21
20
19
Blank
C1
C2
C3
C4
M1
M2
M3
M4
T1
T2
T3
T4
DNA Spike
RNA Spike
B
Raw Ct
40
35
30
25
20
15
10
C1
C2
C3
C4
M1
M2
M3
M4
T1
T2
T3
T4
miR-103
miR-191
miR-451
miR-23a
miR-30c
C
Raw Ct
50
40
30
20
10
0
Positive control
Negative control
miR-103
miR-191
miR-451
miR-23a
miR-30c

**Fig. S5**

**Serum microRNA profiling quality control**

(A) Spike-in control oligonucleotides were detected at similar Ct values across all samples and in the blank control indicating consistent RNA extraction and reverse transcription efficiencies between samples. Similarly, consistency in the Ct values for the RNA and DNA spike controls indicates that PCR-inhibiting contaminants were not co-purified with the RNA samples. (B) miRNAs commonly found in serum samples were present at similar levels across all samples indicating uniform quality. (C) Negative (no-template) control samples amplify >10 cycles later than positive control samples (or are undetected in the case of miR-103).

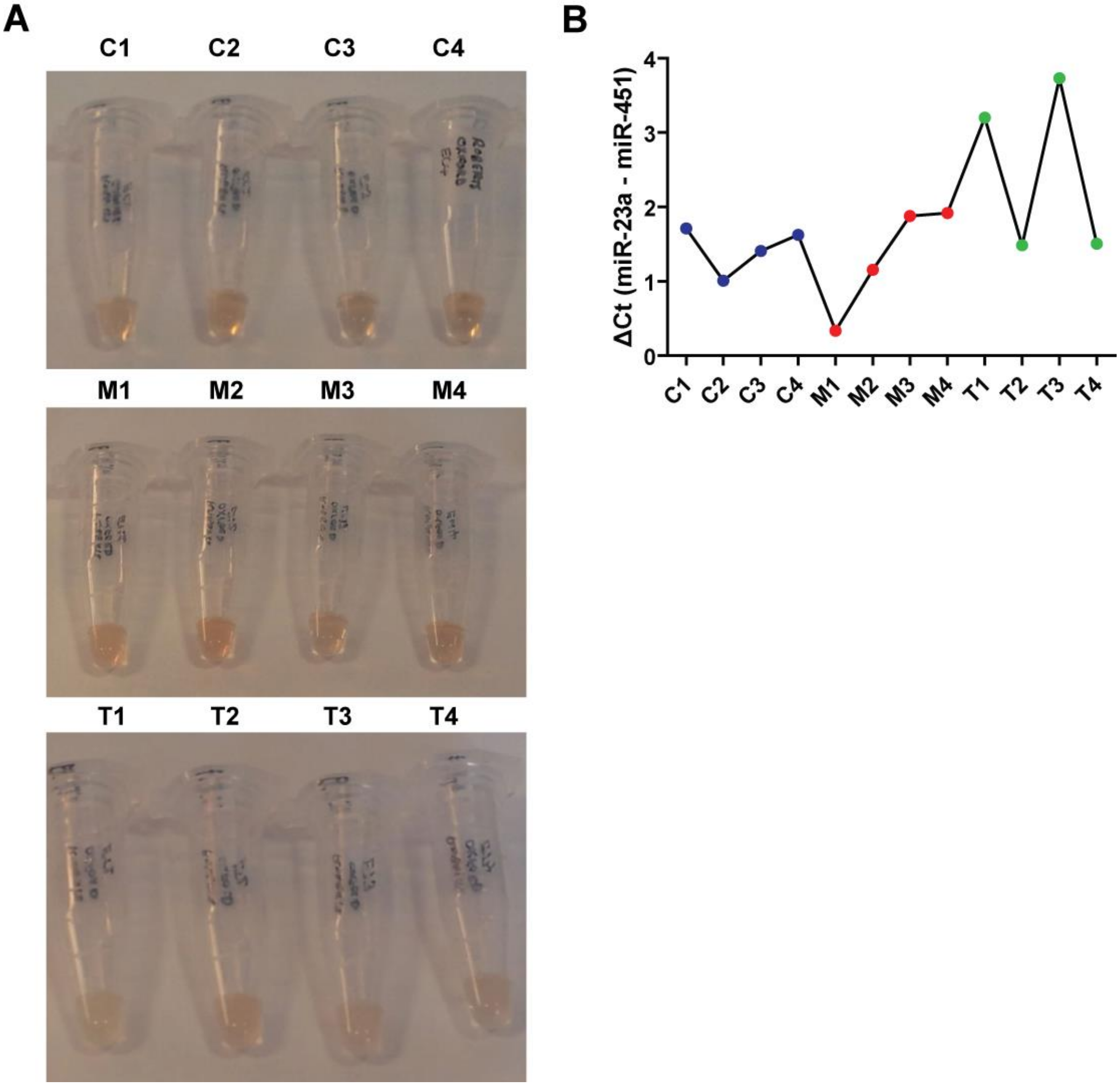


**Fig. S6**

**Assessment of hemolysis**

(A) None of the serum samples used for serum miRNA profiling appeared visibly hemolyzed. The Pip6a-PMO treated *mdx* samples (T1-T4) appear slightly lipemic. (B) The expression ratio of the erythrocyte-enriched miR-451 to non-erythrocyte enriched miR-23a was measured for each serum sample. Samples with ratios greater than 8 are considered hemolyzed.

| | Myog-TA | Myod1-TA | Myf5-TA | miR-1-TA | miR-133a-TA | miR-206-TA | Myog-Diaphragm | Myod1-Diaphragm | Myf5-Diaphragm | miR-1-Diaphragm | miR-133a-Diaphragm | miR-206-Diaphragm | miR-1-Serum | miR-133a-Serum | miR-206-Serum | miR-22-Serum | miR-30a-Serum | miR-193b-Serum | miR-378-Serum |
|---|---|---|---|---|---|---|---|---|---|---|---|---|---|---|---|---|---|---|---|
| Myog-TA | | -0.11 | 0.32 | -0.38 | -0.47 | 0.71 | 0.00 | -0.10 | 0.10 | -0.06 | -0.14 | -0.25 | 0.16 | 0.13 | 0.40 | -0.19 | 0.04 | 0.09 | 0.15 |
| Myod1-TA | -0.11 | | 0.04 | 0.19 | 0.59 | -0.25 | -0.01 | -0.04 | -0.29 | -0.01 | 0.24 | -0.13 | -0.16 | -0.09 | -0.11 | 0.35 | 0.28 | 0.11 | -0.13 |
| Myf5-TA | 0.32 | 0.04 | | -0.75 | -0.32 | 0.36 | -0.64 | -0.61 | -0.45 | 0.59 | 0.44 | 0.42 | -0.29 | -0.21 | 0.04 | -0.25 | -0.05 | -0.14 | -0.20 |
| miR-1-TA | -0.38 | 0.19 | -0.75 | | 0.58 | -0.16 | 0.45 | 0.43 | 0.33 | -0.45 | -0.30 | -0.18 | 0.28 | 0.34 | 0.07 | 0.27 | 0.06 | 0.15 | 0.26 |
| miR-133a-TA | -0.47 | 0.59 | -0.32 | 0.58 | | -0.44 | -0.09 | -0.08 | -0.09 | 0.08 | 0.30 | 0.27 | -0.07 | 0.04 | -0.15 | 0.41 | 0.22 | 0.04 | -0.08 |
| miR-206-TA | 0.71 | -0.25 | 0.36 | -0.16 | -0.44 | | -0.05 | -0.17 | -0.04 | -0.08 | -0.21 | -0.10 | 0.00 | 0.11 | 0.33 | -0.31 | -0.03 | 0.04 | 0.19 |
| Myog-Diaphragm | 0.00 | -0.01 | -0.64 | 0.45 | -0.09 | -0.05 | | 0.93 | 0.54 | -0.89 | -0.70 | -0.66 | 0.04 | 0.04 | -0.14 | 0.03 | -0.12 | 0.05 | -0.04 |
| Myod1-Diaphragm | -0.10 | -0.04 | -0.61 | 0.43 | -0.08 | -0.17 | 0.93 | | 0.65 | -0.79 | -0.61 | -0.68 | 0.17 | 0.09 | -0.08 | 0.09 | -0.10 | 0.07 | 0.06 |
| Myf5-Diaphragm | 0.10 | -0.29 | -0.45 | 0.33 | -0.09 | -0.04 | 0.54 | 0.65 | | -0.25 | -0.29 | -0.21 | 0.10 | 0.09 | 0.04 | -0.26 | -0.39 | -0.09 | 0.11 |
| miR-1-Diaphragm | -0.06 | -0.01 | 0.59 | -0.45 | 0.08 | -0.08 | -0.89 | -0.79 | -0.25 | | 0.89 | 0.79 | -0.32 | -0.36 | -0.18 | -0.31 | -0.13 | -0.39 | -0.30 |
| miR-133a-Diaphragm | -0.14 | 0.24 | 0.44 | -0.30 | 0.30 | -0.21 | -0.70 | -0.61 | -0.29 | 0.89 | | 0.68 | -0.44 | -0.55 | -0.43 | -0.28 | -0.12 | -0.53 | -0.53 |
| miR-206-Diaphragm | -0.25 | -0.13 | 0.42 | -0.18 | 0.27 | -0.10 | -0.66 | -0.68 | -0.21 | 0.79 | 0.68 | | -0.35 | -0.26 | -0.26 | -0.24 | -0.19 | -0.41 | -0.29 |
| miR-1-Serum | 0.16 | -0.16 | -0.29 | 0.28 | -0.07 | 0.00 | 0.04 | 0.17 | 0.10 | -0.32 | -0.44 | -0.35 | | 0.80 | 0.79 | 0.65 | 0.56 | 0.61 | 0.79 |
| miR-133a-Serum | 0.13 | -0.09 | -0.21 | 0.34 | 0.04 | 0.11 | 0.04 | 0.09 | 0.09 | -0.36 | -0.55 | -0.26 | 0.80 | | 0.90 | 0.65 | 0.56 | 0.86 | 0.94 |
| miR-206-Serum | 0.40 | -0.11 | 0.04 | 0.07 | -0.15 | 0.33 | -0.14 | -0.08 | 0.04 | -0.18 | -0.43 | -0.26 | 0.79 | 0.90 | | 0.54 | 0.60 | 0.78 | 0.89 |
| miR-22-Serum | -0.19 | 0.35 | -0.25 | 0.27 | 0.41 | -0.31 | 0.03 | 0.09 | -0.26 | -0.31 | -0.28 | -0.24 | 0.65 | 0.65 | 0.54 | | 0.87 | 0.78 | 0.57 |
| miR-30a-Serum | 0.04 | 0.28 | -0.05 | 0.06 | 0.22 | -0.03 | -0.12 | -0.10 | -0.39 | -0.13 | -0.12 | -0.19 | 0.56 | 0.56 | 0.60 | 0.87 | | 0.75 | 0.56 |
| miR-193b-Serum | 0.09 | 0.11 | -0.14 | 0.15 | 0.04 | 0.04 | 0.05 | 0.07 | -0.09 | -0.39 | -0.53 | -0.41 | 0.61 | 0.86 | 0.78 | 0.78 | 0.75 | | 0.84 |
| miR-378-Serum | 0.15 | -0.13 | -0.20 | 0.26 | -0.08 | 0.19 | -0.04 | 0.06 | 0.11 | -0.30 | -0.53 | -0.29 | 0.79 | 0.94 | 0.89 | 0.57 | 0.56 | 0.84 | |

## Table S1

**Correlation analysis of expression values of miRNAs and myogenic transcription factors in serum and tissues**

| ID | sequence |
|---|---|
| **Myf5-FWD** | CACCTCCAACTGCTCTGAC |
| **Myf5-REV** | ACATGCATTTGATACATCAGGAC |
| **Myf5-PROBE** | FAM-TGCCTGAATGTAACAGCCCT |
| **Myod1-FWD** | GCTCTGATGGCATGATGGAT |
| **Myod1-REV** | GACACAGCCGCACTCTT |
| **Myod1-PROBE** | FAM-ACGACACCGCCTACTACAGT |
| **Myog-FWD** | CGATCTCCGCTACAGAGG |
| **Myog-REV** | CGCGAGCAAATGATCTCCT |
| **Myog-PROBE** | FAM-CCAGTGAATGCAACTCCCACAGC |
| **Tbp-FWD** | AAGAAAGGGAGAATCATGGACC |
| **Tbp-REV** | GAGTAAGTCCTGTGCCGTAAG |
| **Rplp0-FWD** | TGACATCGTCTTTAAACCCCG |
| **Rplp0-REV** | TGTCTGCTCCCACAATGAAG |
| **Rpl10-FWD** | TCATGTCCATCCGAACCAAG |
| **Rpl10-REV** | GCATTAAACTTGGTGAAGCCC |
| **Dmd-exon22-23-FWD** | AACATCAACTTCAGCCATCCA |
| **Dmd-exon22-23-REV** | GAAACTTTCCTCCCAGTTGGT |
| **Dmd-exon22-23-PROBE** | HEX-AGTTTATTCATATGTTCTTCTAGCTTTTGGCAGC |
| **Dmd-exon20-21-FWD** | TGACAATCTGTTGACTTCATCCT |
| **Dmd-exon20-21-REV** | CAGATGACAACTACTGCCGAA |
| **Dmd-exon20-21-PROBE** | FAM-CCAGTCTACCACCCTATCAGAGCCA |

**Table S2**

**Sequences of RT-qPCR Primers and probes used in this study.**
